# A Case Report of a Favorable Outcome of Prolonged Cardiopulmonary Resuscitation in Pregnancy

**DOI:** 10.7759/cureus.89570

**Published:** 2025-08-07

**Authors:** Venus Sulaman, Rumbi Masukume, Phumelele Mthembu, Bakatuamba Pabu

**Affiliations:** 1 Obstetrics and Gynaecology, Rahima Moosa Mother and Child Hospital, Johannesburg, ZAF; 2 Obstetrics and Gynaecology, University of the Witwatersrand, Johannesburg, ZAF; 3 Obstetrics and Gynaecology, Pholosong Regional Hospital, Johannesburg, ZAF; 4 Anaesthesiology, Pholosong Regional Hospital, Johannesburg, ZAF

**Keywords:** cardiac arrest in pregnancy, critical care in obstetrics, perimortem cesarean section, severe respiratory distress syndrome, severe sepsis

## Abstract

Cardiac arrest in pregnancy is a rare event and poses a great risk to the mother and the fetus. A perimortem cesarean delivery (PMCD) is indicated within four minutes of cardiac arrest if the return of spontaneous circulation (ROSC) has not been achieved. This is a case of a 24-year-old pregnant woman who had a cardiac arrest and underwent a PMCD within six minutes. ROSC was achieved after 40 minutes. The patient was discharged home after 53 days without any neurological sequelae. The twins had neonatal encephalopathy and required neonatal ICU admission and cooling. They were discharged on day 12. Early recognition of cardiac arrest and rapid initiation of maternal resuscitation can significantly improve maternal and fetal outcomes. All providers who work closely with pregnant patients should be aware of the latest 2020 American Heart Association guidelines for the management of cardiac arrest in pregnancy.

## Introduction

Cardiac arrest poses a great risk to the mother and the fetus and carries a high mortality rate [1]. This is a rare event, and the incidence of cardiac arrest in pregnancy in Canada is approximately one in 12,500 deliveries, with a survival rate of 71.3% [2]. In a study by Beckett et al. in the UK, the incidence of cardiac arrest in pregnancy was 2.78 per 100,000 maternities, with a case fatality rate of 42% [3].

Perimortem cesarean delivery (PMCD) was first described in 715 BC. Initially, maternal and fetal survival was poor due to a delay in the delivery of the fetus until maternal death was established [4]. The physiological changes in pregnancy cause numerous challenges in the resuscitative measures. Most pregnant women are relatively healthy. The fetoplacental unit increases oxygen consumption by 20%, and in an unwell mother, it causes more compromise [5]. Pregnant patients become hypoxic more rapidly due to reduced functional residual capacity. In cases when the uterine fundus is greater than the umbilicus, perimortem cesarean section can substantially improve the patient’s hemodynamics [1].

In most cases, the survival rate of cardiac arrest in the hospital varies between 0% and 42%. Other co-morbid conditions, such as sepsis, cancer, renal failure, and other chronic illnesses, are associated with poor survival rates. A study by Sandroni et al. shows that the rate of chest compression greater than 80 per minute improves survival rates [6]. The common causes of cardiac arrest in pregnancy are amniotic fluid embolism, hemorrhagic shock, severe pre-eclampsia, congenital or acquired heart disease, and trauma [7,8]. This case presented with severe respiratory distress and required a PMCD, and despite cardiopulmonary resuscitation, she survived without neurological fallout.

## Case presentation

The patient was a 24-year-old woman, para 1 gravida 2, booked at five weeks of gestation, with no known co-morbidities. She was HIV negative, Rhesus blood group positive, and rapid plasma reagin (RPR) negative, with a booking hemoglobin of 12.2. She had a total of five uneventful antenatal visits in her local clinic. No baseline ultrasound was done.

She presented at Pholosong Regional Hospital at 9:44 with a one-day history of acute shortness of breath to the labor ward admission area. She was at 36 weeks of gestation by her last menstrual period (LMP). She appeared in severe respiratory distress, using accessory muscles with a respiratory rate of 40 breaths per minute. Her vital signs showed a blood pressure of 164/114, pulse rate of 160, and saturation of 69% with a face mask receiving 100% oxygen. On examination of the chest, she was noted to have bilateral coarse crackles, and on cardiac examination, S1 and S2 were audible. The abdomen was gravid with a symphysis fundal height of 37 cm, and she had bipedal edema. A quick bedside ultrasound was difficult to perform as the patient was restless. It revealed an intrauterine pregnancy with a positive fetal heart.

An arterial blood gas test on 40% oxygen showed a pH of 7.21 (7.35-7.45), partial pressure of carbon dioxide (PCO2) of 31 (35-45 mmHg), and partial pressure of oxygen (PO2) of 48 (80-100 mmHg). Sodium (Na) level was at 135 (136-146 mmol/L), potassium (K) at 4.1 (3.7-4.7 mmol/L), glucose at 6.2 (3.5-5.4 mmol/L), lactate at 6.7 (0.00-2.0 mmol/L), hemoglobin at 12.6 (12.0-15.0 dL), and base excess at -15.0 (-3.0-3.0 mmol/L), which showed hypoxemia and metabolic acidosis. Our differential diagnosis was a possible peripartum cardiomyopathy, severe preeclampsia with pulmonary edema, or a lower respiratory tract infection.

**Figure 1:**
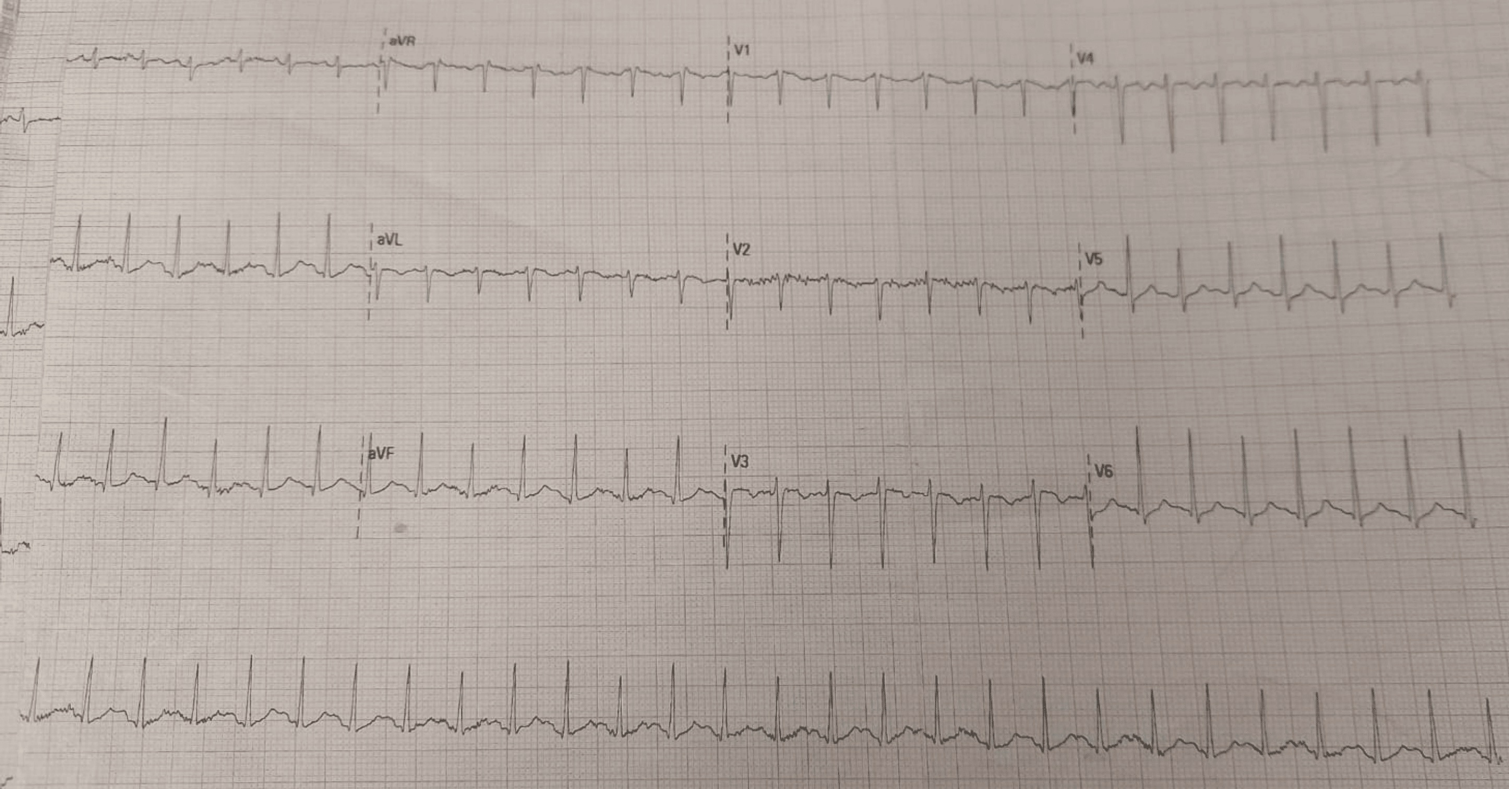
ECG showed sinus tachycardia and no sign of left or right hypertrophy.

The patient was placed in a semi-sitting position on facemask oxygen, 40 mg of Lasix was given intravenously, and 1.2 g of Augmentin was administered. ECG and an urgent medical consultation were done, and an ICU bed was arranged for the patient. Figure 1 demonstrates the ECG showing sinus tachycardia.

**Figure 2:**
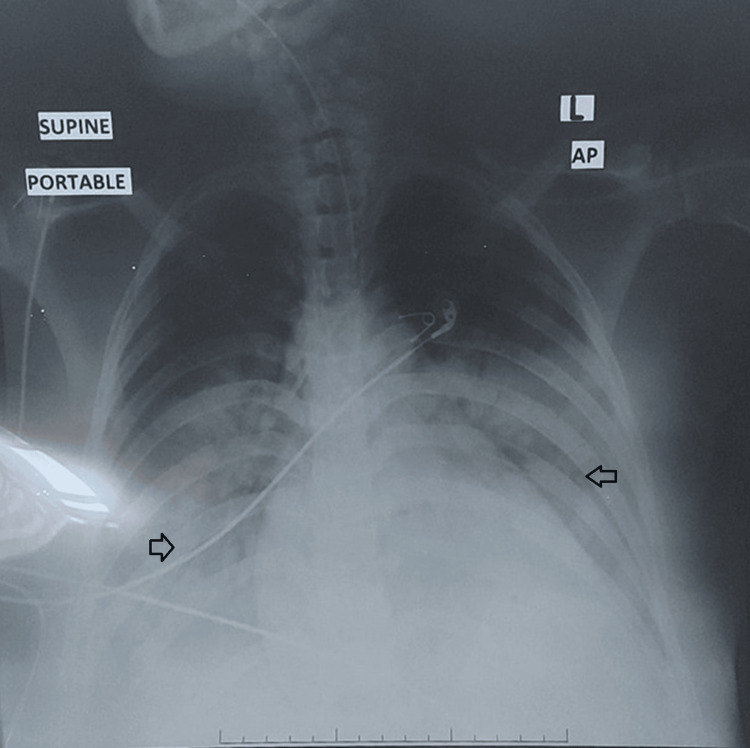
Chest X-ray showed bilateral infiltrates, a normal cardiothoracic ratio, and an endotracheal tube in the correct position.

At 10:25, the anesthesiology team attempted rapid sequence intubation, and 24 mg of Etomidate and 80 mg of succinylcholine were administered. The patient had a cardiac arrest immediately after the intubation. Cardiac resuscitation was started immediately. A perimortem cesarean section was performed six minutes after the resuscitation. Undiagnosed twins were delivered; they were floppy and required neonatal resuscitation by the pediatric team. The first twin was 2350 g with an Apgar score of 6 at 10 minutes. The second twin was 2250 g with an Apgar score of 7 at 10 minutes. Return of spontaneous circulation (ROSC) was achieved after a total of 40 minutes of cardiopulmonary resuscitation. The portable chest X-ray after resuscitation showed bilateral infiltrates and an endotracheal tube in situ (Figure 2).

The patient was maintained on adrenaline infusion and received one unit of packed red blood cells. A subtotal hysterectomy was performed in the ICU due to the unavailability of the theater. The patient had a Glasgow Coma Scale (GCS) score of 10/T the following day, and a CT brain showed no hypoxic brain injuries.

The challenges during the ICU stay were the need for inotropic support and acute renal failure, for which she required four sessions of hemodialysis, and subsequently, the renal function improved. The fluid management of the patient was difficult as she would easily become fluid-overloaded. She had several episodes of hypernatremia, which were managed accordingly. We did not have a cardiology consultant or a cardiac echogram facility in our regional hospital. The case was discussed with the tertiary referral hospital, but there were no available beds.

She was started empirically on Augmentin for five days. The septic markers remained raised, but the blood cultures did not show any growth. Her surgical wound healed well, and the sutures were removed on day 10. There was a 2 cm skin breakdown, which healed with a wound dressing. She subsequently received 10 days of renal-adjusted dose of tazobactam and piperacillin. She was extubated on day 16 of ICU stay, but was re-intubated after three days. She had spiking temperatures, and her wound pus swab showed a growth of *Acinetobacter baumannii*, and the blood cultures had a growth of *Klebsiella pneumoniae* sensitive to ceftazidime.

She had a good response to the culture-directed antibiotics. A tracheostomy was done on day 32 of ICU stay; she coped well and was stepped down to the high care unit. With daily physiotherapy, she was able to mobilize and had multiple reviews by the ENT and speech therapist. The tracheostomy was closed after two weeks, and the patient was transferred to the medical ward in a stable condition. She was discharged home after 54 days of hospital stay.

## Discussion

In cardiac resuscitation, the survival of the patient depends on the location of the collapse and the time of ROSC. A collaboration between the obstetrics, neonatal, emergency, anesthesiology, and intensivists is needed for a successful resuscitation and good maternal and fetal outcomes [7]. The basic principles include the left lateral uterine displacement if the height of the fundus is greater than the umbilicus to relieve the aortocaval compression. Within four minutes of the cardiac arrest, if ROSC has not been achieved, a PMCD must be performed [7]. With regards to the chest compressions, the same hand placement, compression rate, and depth are recommended as in non-pregnant patients; however, the compressions should be conducted a bit higher on the chest as the gravid uterus elevates the diaphragm [9].

This case had a favorable maternal and fetal outcome. The cardiac arrest occurred in the intensive care unit with the presence of the obstetrics, anesthesiology, and intensive care unit teams. Even though the main purpose of the PMCD is to improve maternal circulation, a study by Katz et al. that reviewed 269 cases of maternal cardiac arrest showed that 70% of the infants who were delivered within four minutes of maternal collapse showed no neurological sequelae [10].

In the above case, the PMCD was performed six minutes after the cardiac arrest. A study in Japan on 18 PMCD cases between April 2010 and April 2015 showed 12 women who did not survive or stayed in a persistent vegetative state, and six women who were discharged without major neurological sequelae. The study showed that the median interval time from cardiac arrest to PMCD was significantly shorter in the group with the favorable outcome (p = 0.002) [11].

The patient in this case was in pulmonary edema due to severe pre-eclampsia, and prompt airway management, oxygenation, and endotracheal intubation were performed. A midline incision was done for the PMCD in this case; however, the surgeon can perform any method that ensures rapid access to the abdomen and has the least morbidity for the patient [12].

The decision regarding the termination of resuscitation (TOR) needs to be individualized. A study by Kudu et al. has combined end-tidal carbon dioxide (EtCO2) values to aid in TOR decision-making. In this study, extending resuscitation for 20 minutes with a cut-off EtCO2 of 20.5 mmHg showed ROSC in none of the study patients. The specificity was 100% (95% CI: 63.1-100.0), sensitivity was 20.0% (95% CI: 9.1-35.7), positive predictive value was 100%, and negative predictive value was 20.0% (95% CI: 17.6-22.6) for patients with no ROSC [13].

This patient had no hypoxic brain injury despite the 40-minute duration of the cardiac resuscitation. She had a GCS score of 10/T the following day and was able to follow instructions. Upon discharge, there was no focal neurologic or memory impairment, and she showed a Cerebral Performance Category Scale score of 1. Sepsis is a big challenge in patients with prolonged ICU stay, with the ventilator and central lines, and in this specific case, the PMCD and the subtotal hysterectomy were done in the ICU in a non-sterile setting. Our patient had a good response to the treatment directed by culture and sensitivity.

## Conclusions

Cardiac arrest is a rare complication of pregnancy that all obstetricians should be prepared for, especially given the increasing number of women with high-risk pregnancies. Early recognition of cardiac arrest and rapid initiation of maternal resuscitation can significantly improve maternal and fetal outcomes. All providers who work closely with pregnant patients should be aware of the latest 2020 American Heart Association guidelines for the management of cardiac arrest in pregnancy. Regular simulations and drills can ensure that the team members are ready to act swiftly in times of emergency. Once the return of circulation has been achieved, the management of possible hemorrhage, cardiac failure, and sepsis is crucial to ensure the recovery of the patient.
